# Dzip3 regulates developmental genes in mouse embryonic stem cells by reorganizing 3D chromatin conformation

**DOI:** 10.1038/srep16567

**Published:** 2015-11-16

**Authors:** Daishi Inoue, Hitoshi Aihara, Tatsuharu Sato, Hirofumi Mizusaki, Masamichi Doiguchi, Miki Higashi, Yuko Imamura, Mitsuhiro Yoneda, Takayuki Miyanishi, Satoshi Fujii, Akihiko Okuda, Takeya Nakagawa, Takashi Ito

**Affiliations:** 1 Department of Biochemistry, Nagasaki University School of Medicine; 2 Nagasaki University Graduate School of Biomedical Sciences; 3 Department of Pediatrics, Nagasaki University Hospital; 4 Department of Environmental Science, Nagasaki University; 5 Kyushu Institute of Technology, Fukuoka 820-8502, Japan; 6 Division of Developmental Biology, Saitama Medical School Research Center for Genomic Medicine.

## Abstract

In mouse embryonic stem (mES) cells, ubiquitylation of histone H2A lysine 119 represses a large number of developmental genes and maintains mES cell pluripotency. It has been suggested that a number of H2A ubiquitin ligases as well as deubiquitylases and related peptide fragments contribute to a delicate balance between self-renewal and multi-lineage differentiation in mES cells. Here, we tested whether known H2A ubiquitin ligases and deubiquitylases are involved in mES cell regulation and discovered that Dzip3, the E3 ligase of H2AK119, represses differentiation-inducible genes, as does Ring1B. The two sets of target genes partially overlapped but had different spectra. We found that Dzip3 represses gene expression by orchestrating changes in 3D organization, in addition to regulating ubiquitylation of H2A. Our results shed light on the epigenetic mechanism of transcriptional regulation, which depends on 3D chromatin reorganization to regulate mES cell differentiation.

Embryonic stem (ES) cells are distinguished from other cell types by their unique ability to maintain self-renewal and differentiate into multiple lineages, and they have a complex network of epigenetic pathways for maintaining a delicate balance between these two processes. In this network, common target genes are regulated by complementary and opposing epigenetic activities, and therefore ES cells are poised to differentiate into various types of cells in a short period of time^1^,^2^.

Histone H2A lysine 119 (H2AK119) is a highly conserved residue, and mono-ubiquitylated H2AK119 (ubH2A) plays a role in transcriptional repression. Until now, Ring1A/B, Rnf8, and Dzip3 (also known as 2A-HUB) have been reported to exhibit E3 ligase activity towards H2AK119. Among these proteins, Ring1B, one of the most common subunits of the polycomb repressor complex 1 (PRC1), acts as a repressor of a large number of developmental genes and regulates differentiation mechanisms in ES cells^3^,^4^,^5^. Like PRC1, PRC2 also comprises a large multiprotein complex containing polycomb group (PcG) proteins, which are memory factors involved in heritable silencing of homeotic genes. PcG proteins prepare ES cells for lineage commitment by temporal control of the expression of a key set of developmental genes and are necessary for cell fate transitions. Ring1B is known as a repressor of a large number of developmental genes in ES cells; however, only a subset of Ring1B-bound genes is de-repressed by deletion of Ring1B. This partial de-repression can be explained by functional redundancy with Ring1A, which, like Ring1B, is an E3 ligase targeting H2AK119. However, even double knockout of Ring1A and Ring1B does not lead to the complete removal of ubH2A around the transcription start site (TSS)^6^. This result suggests the existence of additional site-specific factors that are involved in mediating ubH2A modifications and repressing a specific set of genes.

Dzip3 is known to be an E3 ligase targeting H2AK119 and was first identified as an RNA-binding RING-dependent ubiquitin ligase^7^. In C2C12 cells, Dzip3 shows nuclear localization and modulates specific histone modifications, rather than exerting global effects through interactions with Nco-R, HDAC1, and HDAC3^8^. It is of great interest whether Dzip3 contributes to gene regulation in ES cells.

For ES cells to differentiate, they need to switch on developmental genes by regulation of an epigenetic pathway involving deubiquitylases, which remove ubiquitin moieties from H2AK119. Until now, five deubiquitylases (USP3^9^, USP16^10^, USP21^11^, USP22^12^, and 2A-DUB [also known as MYSM1]^13^) have been reported. Recently, USP16 and USP22 were shown to regulate the differentiation process in mouse ES (mES) cells^14^,^15^. However, whether other deubiquitylases also contribute to the gene regulation of ES cells has not been determined.

In this study, we tested whether H2A ubiquitin ligases and deubiquitylases are involved in the regulation of pluripotency and the differentiation process in mES cells and demonstrated that Dzip3 regulates developmental genes in mES cells by reorganizing 3D chromatin conformation.

## Results

### Dzip3 regulates developmental genes

ES cells have a distinct morphology, a small size with little cytoplasm, and tightly packed colonies with round or polygonal borders. Upon differentiation, the cells typically expand and flatten out, often losing their tightly packed appearance, which leads to expansion of the colony. To identify H2A ubiquitin ligase or deubiquitylase activities, which play important roles in mES cells, we examined changes in ES cell morphology after performing siRNA knockdown (KD) of eight proteins: Ring1B (also known as Rnf2), Rnf8, and Dzip3, which are ubiquitin ligases, and USP3, USP16, USP21, USP22, and Mysm1, which are deubiquitylases^16^,^17^,^18^.

**Figure 1:**
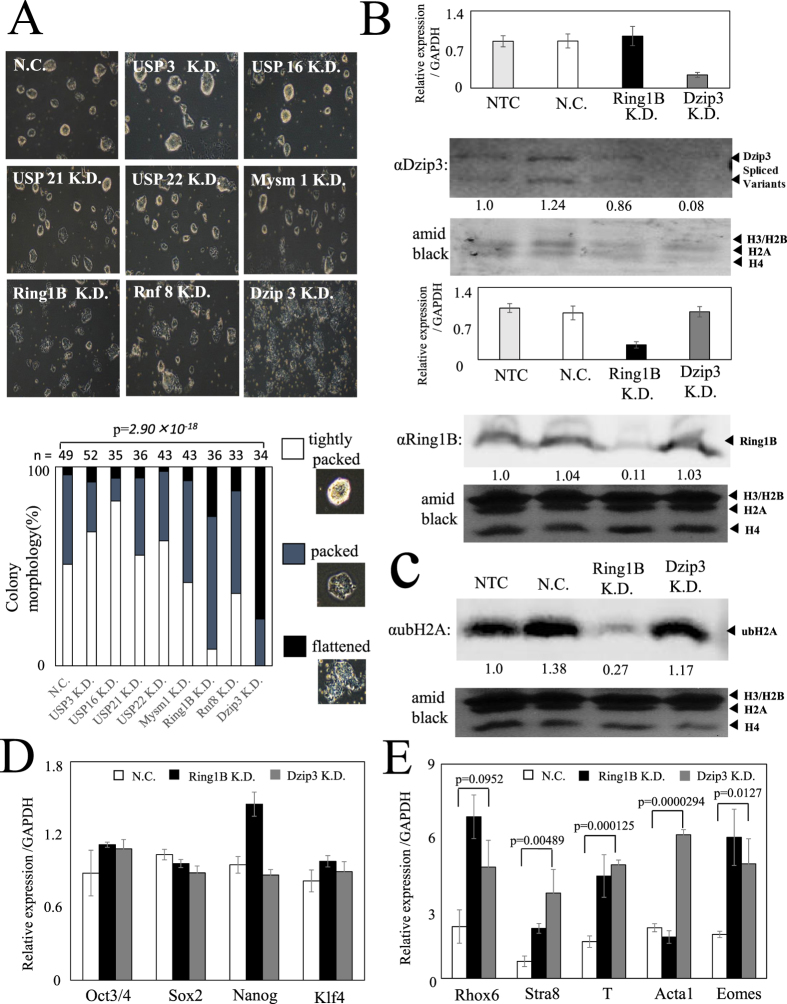
Knockdown of Dzip3 results in a decrease in the percentage of tightly packed cell colonies and upregulates differentiation-inducible gene expression. (**A**) The morphology of mES cells 72 h after siRNA transfection. Knockdown (KD) of Ring1B and Dzip3 resulted in a decrease in the percentage of tightly packed cell colonies. There were three categories of colony morphology: “tightly packed” (observed in pluripotency); “flattened” (observed in differentiation); and “packed” (intermediate between tightly packed and flattened). The n value represents the number of colonies classified. Statistical significance was assessed using the HYPGEOMDIST function (*P* = *2.90* × *10*^−*18*^, negative control [NC] versus Dzip3 KD samples). (**B**) The efficiency of knockdown was evaluated in mES cells (serum + LIF) transfected with the indicated specific and control siRNAs. RT-qPCR analysis was performed to document the efficiency of Dzip3 siRNA and Ring1B siRNA knockdown to diminish endogenous Dzip3 and Ring1B. Values (normalized to the corresponding values of the internal control gene GAPDH) are the mean ± SEM of three independent experiments. Protein levels were determined by western blotting using the indicated antibodies. Equal loading was confirmed by Amido Black staining. The two major bands correspond to the alternatively spliced versions of Dzip3. Full-length blots are presented in Supplementary Fig. 2a,b. (**C**) The ubH2A level was determined by western blotting. Full-length blots are presented in Supplementary Fig. 2c. (**D**) The relative expression levels of pluripotent marker genes in NC, Ring1B KD, and Dzip3 KD mES cells. (**E**) The relative expression levels of differentiation-inducible genes adjusted with GAPDH in NC, Ring1B KD, and Dzip3 KD mES cells. Statistical significance was assessed by two-tailed Student’s *t*-test. NTC, no-treatment control. Error bars, standard deviation.

First, we focused on morphological changes under pluripotent conditions (serum + LIF)^19^. KD of Ring1B and Dzip3 resulted in a decrease in tight packing of the cells and led to an expansion of the colony (Fig. 1A and Supplementary Fig. 1b). KD of Dzip3 and Ring1B was confirmed by RT-qPCR analysis and western blotting (Fig. 1B and Supplementary Fig. 2a), and the amount of H2A ubiquitylation (ubH2A) in the cell was determined by western blotting. The total amount of ubH2A in the cell decreased after Ring1B KD but not after Dzip3 KD (Fig. 1C), which suggests that Dzip3 functions by modulating a specific histone modification in the promoter region, rather than by global effects.

To determine the effect on gene transcription of Dzip3 KD in mES cells, we investigated the gene expression of pluripotency markers by RT-qPCR (Fig. 1D). However, transcriptional changes in these markers were not apparent. The transition from a pluripotent stem cell to a committed cell type is accompanied by stable silencing of pluripotency genes and activation of lineage-specific genes. We therefore next investigated lineage-specific gene expression after Dzip3 KD in mES cells. The transcriptional levels of several lineage-specific genes, such as *Rhox6*, *Stra8*, *T* (encoding the Brachyury protein), *Acta1*, and *Eomes,* were upregulated by Dzip3 KD. Significant numbers of genes were upregulated by simultaneous KD of Dzip3 and Ring1B (Fig. 1E). These results suggest that Dzip3, together with Ring1B, represses lineage-specific gene expression in mES cells.

### Whole-transcriptome sequencing analysis shows overlap of Dzip3 and Ring1B target genes

To obtain insight into whole-transcriptome effects after Dzip3 KD in mES cells, we performed RNA-seq analysis. Lineage-affiliated gene expression is strongly repressed when mES cells are cultured in the presence of the two inhibitors PD0325901 and CHIR99021 (both components of 2i medium) plus LIF compared with culturing in the presence of fetal bovine serum (FBS) plus LIF. These results indicate that, in the presence of FBS plus LIF, the mES cell is in a metastable state rather than in a state exhibiting the inherent properties of pluripotent cells^20^,^21^. We confirmed that, after Dzip3 KD, the changes in colony morphology and expression levels of pluripotency markers were comparable between ES cells cultured with 2i medium plus LIF and serum plus LIF (data not shown). Therefore, we decided to perform whole-transcriptome sequencing analysis with cultured mES cells under 2i-medium-plus-LIF conditions.

**Figure 2:**
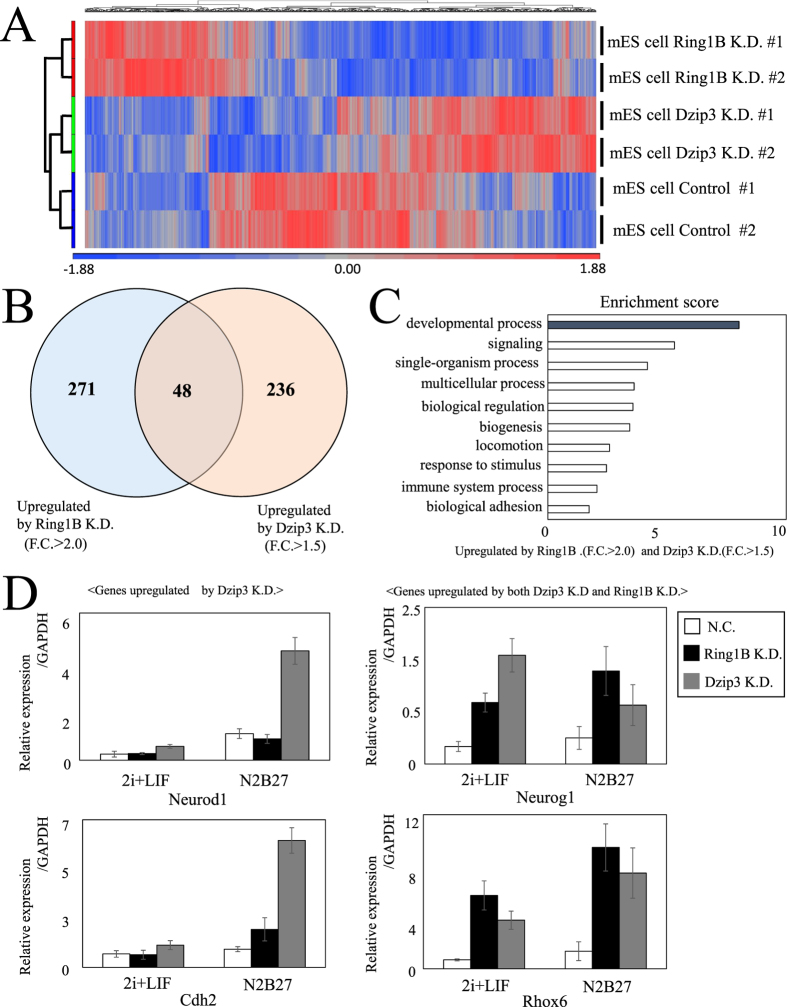
Dzip3 and Ring1B redundantly regulate differentiation-inducible genes. (**A**) Heat map of gene expression in negative control (NC), Ring1B knockdown (KD), and Dzip3 KD mES cells in biological duplicate RNA-seq samples. Blue and red indicate down- and upregulated genes, respectively. (**B**) Venn diagram representing the genes upregulated by Ring1B KD and Dzip3 KD cells. The numbers represent the numbers of genes upregulated (>1.5 or >2.0 fold) by KD of either one of the two proteins or by KD of both proteins. (**C**) Gene ontology (GO) analysis of genes upregulated by both Ring1B KD and Dzip3 KD. (**D**) RT-qPCR results for major developmental genes (*Neurod1*, *Rhox6, Cdh2*, *Neurog1*). Error bars, standard deviation.

Whole-transcriptome sequencing revealed that KD of either Dzip3 or Ring1B altered expression levels of a significant number of genes, but the genes affected by the former were somewhat different from those affected by the latter (Fig. 2A and Supplementary Fig. 3a). However, there was a substantial set of genes whose expression was affected by both proteins. A Venn diagram (Fig. 2B) represents the overlap between the genes de-repressed by Ring1B KD and those by Dzip3 KD, showing that about one fifth of the genes whose expression was derepressed by Dzip3 KD were also derepressed by Ring1B KD (Fig. 2B). To gain insight into the molecular functions of genes that were repressed by both Dzip3 and Ring1B, we conducted gene ontology (GO) classification analyses, and the results suggest that the common targets of Dzip3 and Ring1B are mostly participants in developmental processes (Fig. 2C).

Some developmental genes that were upregulated by Dzip3 KD or Ring1B KD were selected based on RNA-seq results and validated by RT-qPCR (Fig. 2D). *Neurod1* and *Neurog1* are expressed in neuroectoderm, *Rhox6* is involved in primordial germ cell differentiation, and *Cdh2* is a neural cadherin. *Neurod1* and *Cdh2* were mainly upregulated by Dzip3 KD, while *Neurog1* and *Rhox6* were upregulated by both Dzip3 and Ring1B KD. N2B27 differentiation conditions caused a typical flattened morphology of ES cell colonies, but no noticeable changes were evident due to Dzip3 KD under differentiation-inducing conditions (data not shown). However, we found that, for some genes, Dzip3 KD not only derepressed their expression under pluripotent conditions but also elevated the magnitude of differentiation-associated induction of gene expression under differentiation-inducing conditions. These results suggest that Dzip3 represses developmental genes at the lineage-commitment stage in mES cells.

### Genome-wide ChIP analysis shows relationships between Dzip3- and Ring1B-binding loci

**Figure 3:**
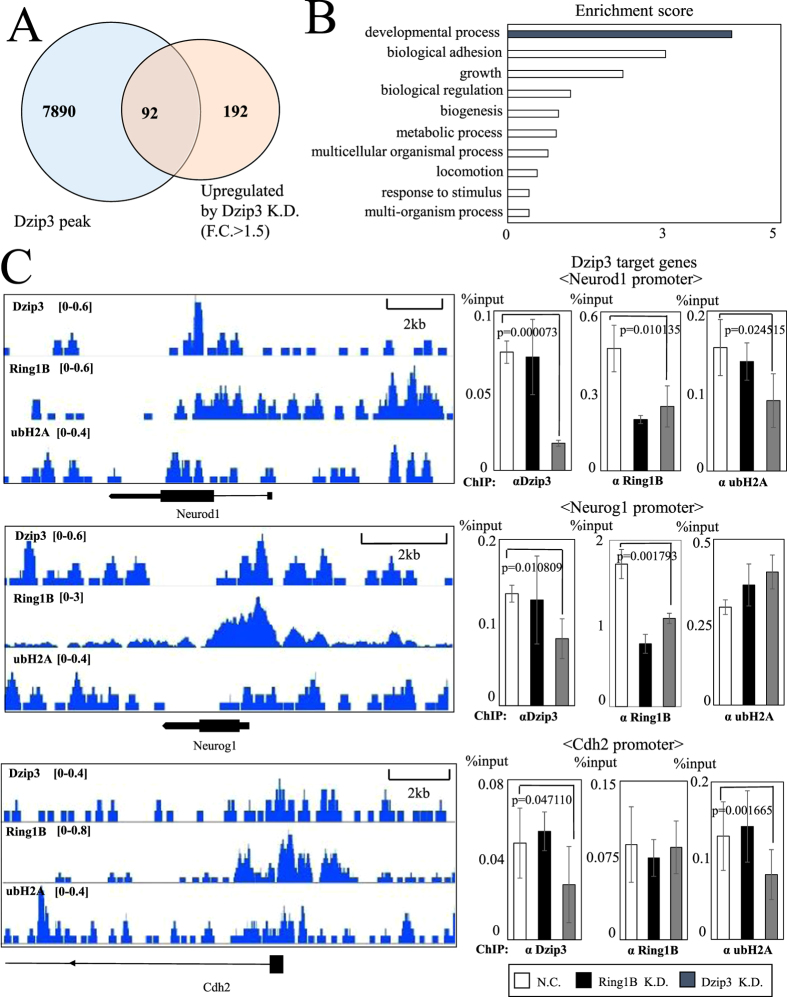
Developmental genes are regulated by Ring1B, Dzip3, and H2A ubiquitylation. (**A**) Venn diagram representing the overlap between genes having Dzip3 peaks around the transcription start site (TSS) by ChIP-seq analysis and upregulated genes in Dzip3 KD cells by RNA-seq analysis. The number in the overlapped region represents the total number of genes exhibiting both. (**B**) Gene ontology (GO) analysis of Dzip3 target genes. (**C**) Left panel: ChIP-seq signal profiles of Dzip3, Ring1B, and ubH2A around the promoter region. Right panel: ChIP-qPCR analysis of the promoter region for negative control (NC), Ring1B KD, and Dzip3 KD mES cells. Statistical significance was assessed using a two-tailed Student’s t-test. Error bars, standard deviation.

To determine the genome-wide locations of Dzip3 and Ring1B binding sites and the positions of ubH2A modification in a genome-wide manner, we performed ChIP-seq with anti-Dzip3, anti-Ring1B, and anti-ubH2A antibodies under pluripotent conditions (2i + LIF). A Venn diagram was used to represent the 92 genes that overlapped between the set of genes bound by Dzip3 around their transcription start sites (TSS) and the set of genes upregulated by Dzip3 KD (Fig. 3A).

To discover the spectrum of genes that are directly regulated by Dzip3 in mES cells, we analyzed these genes based on their gene ontology (GO) classification, which suggested that Dzip3 regulates developmental processes (Fig. 3B).

Next, we validated the ChIP-seq results by designing primers against the promoter regions of important developmental genes and performing ChIP-qPCR on these genes. We confirmed Dzip3 occupancy by comparing the Dzip3 signal around the promoter of *Neurod1*, *Neurog1*, *Cdh2* and other genes in mES cells with control (NC) cells and cells subjected to Dzip3 KD (Fig. 3C) Unexpectedly, Ring1B was also enriched at *Neurog1* loci. Moreover, Ring1B occupancy levels were decreased not only by Ring1B KD but also by Dzip3 KD. These results suggest that Dzip3 plays a role in localization of Ring1B and regulates gene expression together with Ring1B. Furthermore, ubH2A modification levels were not appreciably altered by Dzip3 KD around the promoter region of *Neurog1*, although an apparent decline was evident at the *Cdh2* and *Neurod1* loci. This result suggests that Dzip3 has the potential to repress gene expression not only by ubiquitylating H2A but also by other mechanisms.

### Dzip3 regulates developmental genes by reorganizing 3D chromatin conformation

**Figure 4:**
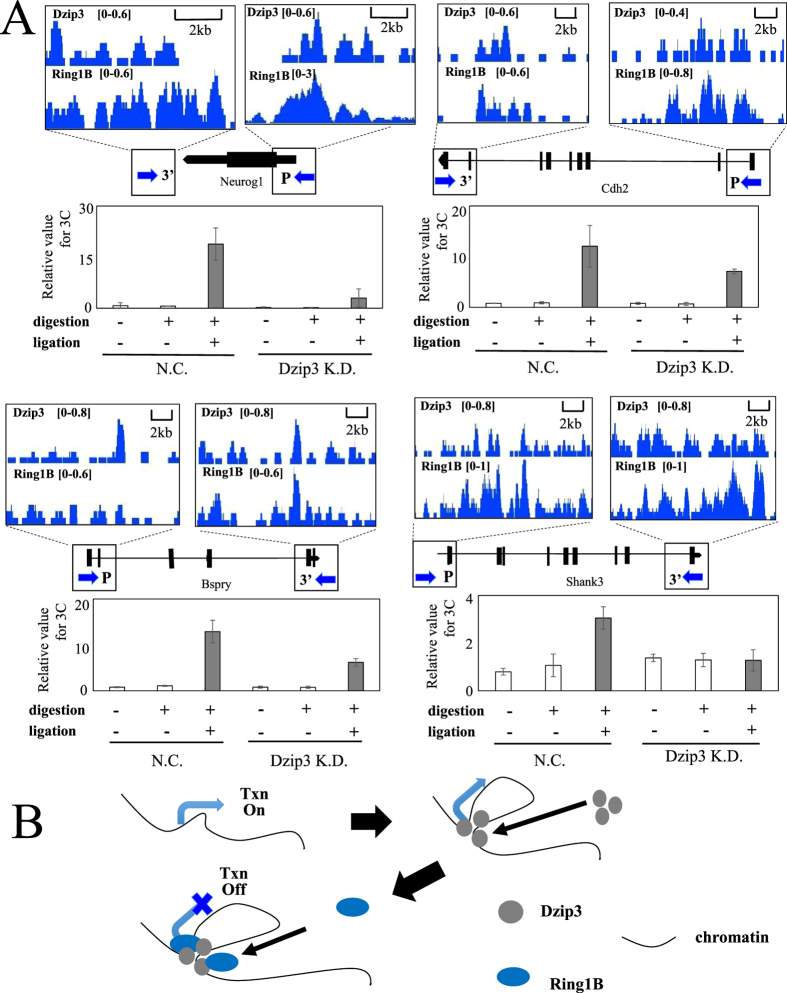
Dzip3 regulates developmental genes by reorganizing 3D chromatin conformation. (**A**) 3C-qPCR analysis of four Dzip3 target genes. Upper panel: ChIP-seq signal profile of target genes. The arrows indicate the primer regions for 3C-qPCR. Lower panel: 3C-qPCR results. Chromatin from Dzip3 knockdown (KD) and negative control (NC) cells was treated with or without digestion and ligation, and the resulting samples were analyzed by qPCR. ChIP-seq signal profile of target genes on a whole-gene scale are presented in Supplementary Fig. 4a. (**B**) Models for transcriptional repression by Dzip3, which promotes interactions between the promoter and regions distal to its binding site. These changes in 3D chromatin structure repress transcription redundantly with Ring1B. Txn, transcription. Error bars, standard deviation.

To explore the molecular mechanisms by which Dzip3 represses gene expression, we focused on those of its target genes around which significant Dzip3 binding sites exist. A recent study revealed that about 25% of Ring1B-bound genes in ESCs possess prominent Ring1B binding sites (RBS) outside of their promoter regions (TSS ± 4 kb) and that Ring1B represses target genes by orchestrating 3D chromatin structural changes^22^. We hypothesized that Dzip3 also participates in regulating target genes by reorganizing 3D chromatin conformation. To assess the validity of this hypothesis, we performed a chromosome conformation capture (3C) assay. We first designed PCR primers for the 3C assay around the promoter region and the 3’ end of the gene, as indicated by arrows (Fig. 4A and Supplementary Fig. 4a). Undigested and unligated samples were prepared as negative controls. For *Neurog1*, *Cdh2*, *Bspry*, and *Shank3*, we detected signals implicating an interaction between the promoter region and the 3’ end of the gene in the presence of the restriction enzyme Mse I and T4 DNA ligase, but not in their absence. Furthermore, the signal indicating chromatin conformation was decreased by Dzip3 KD (Fig. 4A and Supplementary Fig. 5a). Therefore, we suggest that Dzip3 regulates gene expression by changing the local chromatin conformation.

## Discussion

The results of this study indicate that Dzip3 represses differentiation-inducible genes in mES cells, possibly by regulating ubH2A and orchestrating changes in 3D chromatin structure. ubH2A has been reported to inhibit transcription initiation by interfering with H3K4 methylase, inhibiting the elongation of transcription by RNA pol II, or facilitating the recruitment of PRC2 and H3K27 trimethylation^8^,^11^,^23^. It was also previously reported that Dzip3 depletion didn’t result in significant changes in the general level of ubH2A and that Dzip3 functions by modulating histone modifications at specific sites rather than globally^8^. In our study, KD of Dzip3 was not accompanied by a global decline in ubH2A modification, but instead, changes were restricted to gene promoters. However, our data demonstrated that binding of Dzip3 did not necessarily lead to a decline in ubH2A levels in some gene promoters, and therefore our results suggest that Dzip3 is also able to repress gene expression levels without ubiquitylating H2A. We assume that the ability of Dzip3 to reorganize 3D chromatin conformation, which was uncovered in this study, is closely linked to this H2A ubiquitylation-independent gene repression.

Recent studies on the distribution of PcG proteins in the mammalian genome revealed their preferential association with genes encoding developmental regulators, which exhibit dynamic changes in their spatiotemporal expression during development^24^. Given the results in this study, it is tempting to speculate that Dzip3 substantially contributes to such preferential Ring1B binding at the promoters of genes encoding developmental regulators.

PcG-mediated gene-silencing mechanisms remain elusive; however, it is obvious that there are several distinct mechanisms operating, which include modifications of histone tails (histone H3K27 trimethylation and H2AK119 mono-ubiquitination), condensation of chromosome segments, and mediation of the interactions between topologically distant regulatory elements. In this study, we propose that Dzip3 represses gene expression in conjunction with Ring1B by ubiquitylating ubH2A and inducing conformational changes in 3D chromatin structure (Fig. 4B).

## Methods

### Cell culture

E14 mES cells were cultured under feeder-free conditions on 0.5% gelatin-coated dishes with mouse embryonic stem cell (ESC) medium consisting of knockout DMEM (Gibco), 10% FBS (Gibco), 2 mM L-glutamine (Merck Millipore), 1% nonessential amino acids (Merck Millipore), 0.3 mM β-mercaptoethanol (Nacalai Tesque), and 1000 U/ml LIF (Merck Millipore) at 37 °C and 5% CO_2_. For the differentiation assay, mES cells were cultured in mouse ESC medium without LIF and supplemented with 0.5 μM retinoic acid^25^. To change from serum + LIF to 2i + LIF conditions, E14 mES cells cultured with serum + LIF were subcultured in 2i + LIF medium (N2B27 medium [Life Technologies] with 1 μM PD0325901 and 3 μM CHIR99021 [Stemgent], together known as 2i medium, and 1000 U/ml LIF). After several passages, the adapted cells were used for study. For the differentiation assay under 2i + LIF conditions, mES cells were cultured without 2i + LIF^20^,^21^.

### RNA interference

mES cells (1.0 × 10^5^) were seeded in 10-cm plates (previously coated with 0.5% gelatin) with mouse ESC medium. Forty-eight hours after seeding, the cells were transfected using Lipofectamine RNAiMAX reagent (Life Technologies) and Opti-MEM I Reduced Serum medium (Life Technologies), according to the manufacturer’s protocol. The targeted siRNA or control siRNA (300 pmol, Life Technologies) were used for transfection. Cells were cultured with a change of medium every 24 hr and collected after 72 hr of siRNA transfection. The siRNA sequence information is shown in Table S1.

### RNA extraction and RT-qPCR

Total RNA was isolated using ISOGEN II reagent (Nippon Gene), according to the manufacturer’s protocol. cDNA was created from 0.5 μg total RNA using an oligo(dT) primer (Life Technologies), random hexamers (Takara), and M-MuLV reverse transcriptase (NEB). Real-time RT-PCR using reagents containing SYBR green was performed with an ABI PRISM 7900HT instrument (Applied Biosystems). Expression levels were compared with known standard samples and normalized to GAPDH. Primer sequences are shown in Table S2.

### Statistical analysis

Statistical analysis was performed using formulae provided in Microsoft Excel. The frequency of flattened colonies within the population of Dzip3 KD colonies was compared with the negative control (NC) colonies using a hypergeometric distribution analyzed using the HYPGEOMDIST function in Excel. Student’s *t-*test was used to determine the significance level under the assumptions of two separate means with equal variance. The error bars represent the standard deviation (SD) of three independent experiments.

### RNA-seq

Total RNA was used for preparing an RNA-seq library. RNA quality was checked using the Agilent RNA 6000 Nano kit with an Agilent 2100 Bioanalyzer instrument (Agilent Technologies). RNA-seq libraries were prepared using the TruSeq Stranded mRNA LT Sample Prep kit (Illumina) and sequenced with the MiSeq system (Illumina). Samples were sequenced to a depth of approximately 3 million uniquely mapped reads per sample. Sequences were aligned to the mouse MM9 reference genome with the Illumina Analysis Pipeline, allowing one mismatch. Reads that could be uniquely mapped to a gene were used to calculate the expression level. Accordingly, the gene expression level was quantified by the number of uniquely mapped reads per kilobase of exon per million mapped reads (RPKM). To evaluate the correlation between the RNA-seq duplicate sample data sets, scatter plots were created using the Partek® Genomics Suite. Sequence and gene ontology (GO) analysis were performed with the Partek® Genomics Suite. Significantly enriched GO functional groups were defined as having an enrichment score equal to or greater than 3 (P value < 0.05), and each functional group was assigned with a GO enrichment score calculated using Fisher`s exact test. All RNA-seq data can be found online in the NCBI GEO SuperSeries GSE71884.

### Heat map construction and hierarchical clustering

Heat map construction and hierarchical clustering of the gene expression profiles were performed using the Partek® Genomics Suite. Heat maps for each sample (negative control [NC], Dzip3 KD and Ring1B KD) were normalized by calculating the RPKM based on the sum of all reads found in the exon regions of that gene. One-way analysis of variance (ANOVA) was used to identify differentially expressed genes. By setting P < 0.1 and fold-change (FC) settings FC > 1.5 or FC > 2, we obtained lists of differentially expressed genes between NC and Dzip3 KD or NC and Ring1B KD cells. Hierarchical clustering of NC, Dzip3 KD, and Ring1B KD cells was performed using 894 genes that varied significantly among each groups with a statistical P value < 0.1. We subtracted the mean of the gene expression levels in the six paired samples, normalized each row in the data table, calculated the distance using Pearson correlation, and then used a “pairwise average-linkage” hierarchical clustering method for clustering. The scale was the standardized RPKM value.

### Generation of antibodies

Antibodies against mouse Dzip3 (mDzip3) and mouse Ring1B (mRing1B) were prepared by immunizing rabbits with GST-fusion proteins encoding amino acids 2–80 of mDzip3 or 149–224 of mRing1B. Anti-ubH2A antibodies were prepared by immunizing rabbits with ubiquitylated H2A peptide (CAVLLPK [branched RLRGG-K] TESHHK). After several immunizations, sera were collected, and the specific antibody was purified, as described previously (Nakagawa *et al.*, 2008).

### Western blotting

Lysates were prepared using a sodium dodecyl sulfate (SDS) sample buffer, separated by SDS-PAGE (7% or 12.5%), transferred to a nitrocellulose membrane (Bio-Rad), and blocked with 3% BSA in TBST (TBS containing 0.05% Tween-20). To detect Ring1B, Dzip3, or histone H2A (mono-ubiquitylated K119), membranes membranes were incubated with anti-mRing1B, anti-mDzip3, or anti-ubH2A antibodies, followed by Alexa 647-fused Protein A (Molecular Probes). Fluorescence signals were captured by employing a Typhoon™ FLA 9000 imager (GE Healthcare) to scan the probed membranes, with the PMT setting set at 1000 V.

### Nuclear extraction and immunoprecipitation

For nuclear extraction, cells were washed with PBS and incubated on ice for 30 min in buffer (10 mM Tris-HCl, pH 7.9, 10 mM KCl, 1.5 mM MgCl2, 1 mM DTT, 1 mM PMSF, 1 mM sodium metabisulfite). Next, cells were homogenized and centrifuged to pellet the nuclei, which were then suspended with buffer (25 mM HEPES-KOH, 0.02 mM EDTA, 10% glycerol, 0.01% NP40, 0.2 M KCl), sonicated (12% of maximum power, 10 sec, 3 cycles), and the supernatant collected as a nuclear extract. For immunoprecipitation, the supernatant was incubated with anti-mRing1B or anti-mDzip3 antibodies or purified rabbit IgG as negative control at 4 °C overnight. To precipitate the immune complexes, Protein A Sepharose 4 Fast Flow (GE Healthcare) was added, and the vessel rotated at 4 °C for 1 hr. The beads were washed, and the immune complexes were eluted in 50 mM glycine.

### ChIP-qPCR

mES cells cultured in 2i + LIF were collected, fixed for 10 min in 1% formaldehyde, neutralized with glycine (0.124 M), and stored at –80°C until use. Fixed cells were washed at 4 °C for 10 min in solution I (10 mM HEPES-KOH, 10 mM EDTA, 0.5 mM EGTA, 0.75% Triton X-100), followed by 10 min in solution II (10 mM HEPES-KOH, 1 mM EDTA, 0.5 mM EGTA, 200 mM NaCl), resuspended in lysis buffer (25 mM Tris-HCl, 5 mM EDTA, 150 mM NaCl, 1% Triton X-100, 0.1% SDS, 0.5% Na-deoxycholate), and sheared using a Picoruptor (15 cycles, 30 sec on/30 sec off; Nippon Gene). Lysates were centrifuged to collect the supernatant containing solubilized nucleosomes. The genomic DNA for ChIP-qPCR assay input was purified from the supernatant by reverse cross-linking, phenol-chloroform extraction, and ethanol precipitation and verified to contain genomic DNA fragments with an approximate size of 100–300 bp by 2% agarose gel electrophoresis. The concentration of DNA was measured using a Nanodrop ND-1000 spectrophotometer (Thermo Scientific). For each immunoprecipitation, the supernatant containing ~20 μg of DNA was incubated with anti-mRing1B, anti-mDzip3, or anti-ubH2A antibodies or purified rabbit IgG as negative control at 4 °C overnight. To precipitate the immune complexes, Protein A Sepharose 4 Fast Flow (GE Healthcare) was added, and the vessel rotated at 4 °C for 1 hr. The beads were washed with RIPA buffer (50 mM Tris, pH 8.0, 150 mM NaCl, 1 mM EDTA, 1.0% NP-40, 0.5% Na-deoxycholate, 0.1% SDS), high-salt buffer (50 mM Tris, pH 8.0, 1 mM EDTA, 500 mM NaCl, 1% NP-40, 0.5% Na-deoxycholate, 0.1% SDS), and LiCl buffer (50 mM Tris, pH 8.0, 250 mM LiCl, 1 mM EDTA, 1.0% NP-40, 0.5% Na-deoxycholate). The beads were incubated at 37 °C for 30 min in Tris-EDTA buffer with added RNase (50 μg/ml final concentration). For reverse cross-linking and elution, the beads were incubated at 37 °C for 4 hr and 65 °C for 12 hr in elution buffer (0.5% SDS, 50 mM Tris-HCl, 10 mM EDTA, proteinase K [200 μg/ml final concentration]). DNA from the eluates was purified by phenol-chloroform extraction and ethanol precipitation. Quantitative PCR was performed using the KAPA SYBR FAST qPCR kit (Kapa Biosystems) with an ABI PRISM 7900HT instrument (AQ method). To generate standard curves, duplicate samples for 10%, 1%, and 0.1% of the input DNA were used. The mean percentage input and SD were calculated from triplicate scores of immunoprecipitations relative to the input DNA. Primer sequences are shown in Table S2.

### ChIP-seq

ChIP samples were prepared with a modification of our ChIP-qPCR method. Briefly, mES cells cultured in 2i + LIF were fixed with 1% formaldehyde and processed by fragmentation with micrococcal nuclease (Sigma-Aldrich). For fragmentation, fixed samples were washed with lysis buffer (50 mM HEPES-KOH, 140 mM NaCl, 1 mM EDTA, 10% glycerol, 0.5% NP-40, 0.25% Triton X-100), resuspended in micrococcal nuclease buffer (15 mM Tris-Hcl, 5 mM MgCl_2_, 1 mM CaCl_2_, 25 mM NaCl), and incubated with micrococcal nuclease at 37 °C for 20 min. For chromatin extraction, samples were sonicated (20% of maximum power, 12 sec.) in lysis buffer #2 (10 mM Tris-HCl, 100 mM NaCl, 1 mM EDTA, 0.5 mM EGTA, 0.1% Na-deoxycholate, 0.5% N-lauroylsarcosine) and rotated in 1% Triton X-100 to quench the sarkosyl. The supernatant was collected as chromatin. Input DNA fragmentation was confirmed by electrophoresis, and the amount of nucleotide was measured with a Nanodrop ND-1000 spectrophotometer (Thermo Scientific). The same amount of input DNA was mixed with antibody (anti-mRing1B, anti-mDzip3, or anti-ubH2A antibodies or purified rabbit IgG as negative control) and sepharose A beads to react and rotated at 4 °C overnight. The beads were washed with RIPA buffer (50 mM HEPES-KOH, 500 mM LiCl, 1 mM EDTA, 1.0% NP-40, 0.7% Na-deoxycholate) and incubated at 37 °C for 30 min in TE with added RNase (50 μg/ml final concentration). For reverse cross-linking and elution, the beads were incubated at 65 °C for 10 hr in elution buffer (1% SDS, 50 mM Tris-HCl, 10 mM EDTA). Elution samples were incubated at 55 °C for 2 hr after adding proteinase K (200 μg/ml final concentration). DNA from the eluates was purified by phenol-chloroform extraction and ethanol precipitation. ChIP-seq libraries were prepared from ChIP samples using the ChIP-Seq Sample Prep kit (Illumina). The resulting libraries were sequenced with the MiSeq sequencing system (Illumina). Samples were sequenced to a depth of approximately 30 million uniquely mapped reads per sample. Sequences were aligned to the mouse MM9 reference genome with the Illumina Analysis Pipeline, allowing one mismatch. To compensate for differences in sequencing depth and mapping efficiency, the data were normalized using MACS2 software, resulting in signal per million reads (SPMR). Peak calling was also performed using the Partek® Genomics Suite™ (PGS), with a statistical false discovery rate (FDR) value of 0.1. The genes having Dzip3 peaks within 100 kb upstream or 100 kb downstream of their TSS were determined using the PGS command “Find nearest genomic features”. GO classifications were also assigned using PGS. To visualize the ChIP-seq results, we used the Integrative Genomics Browser (BioViz). All ChIP-seq data can be found online in the NCBI GEO SuperSeries GSE71884.

### Two-step cross-linking

For chromatin preparation, cells were washed on a plate three times with PBS and incubated with 2 mM disuccinimidyl glutarate (DSG, Thermo Scientific) in PBS for 30 min at room temperature. ES cells were washed in PBS three times, fixed for 10 min in 1% formaldehyde, neutralized with glycine (0.124 M), and stored in –80°C until use. All subsequent ChIP-qPCR steps were performed as described above.

### 3C-qPCR

3C assays were performed as described previously^26^. Briefly, mES cells cultured in 2i + LIF were fixed with 1% formaldehyde and resuspended in lysis buffer. Nuclei were treated with Mse I restriction enzyme (NEB) and reacted with T4 DNA ligase (NEB). The genomic DNA was then reverse cross-linked and purified by phenol-chloroform extraction and ethanol precipitation. Each purified DNA concentration was quantified using a Nanodrop ND-1000 spectrophotometer to adjust the qPCR concentration to 50 ng/μl. Quantitative PCR was performed using the KAPA SYBR FAST qPCR kit with the ABI PRISM 7900HT instrument (AQ method). To generate standard curves, duplicate samples with 30-, 150-, and 750-fold diluted DNA were used. Relative values for 3C were normalized using qPCR values of a GAPDH allele lacking restriction sites within its PCR product. Primer sequences are shown in Table S2.

## Additional Information

**How to cite this article**: Inoue, D. *et al.* Dzip3 regulates developmental genes in mouse embryonic stem cells by reorganizing 3D chromatin conformation. *Sci. Rep.* **5**, 16567; doi: 10.1038/srep16567 (2015).

## Supplementary Material

Supplementary Information

## References

[b1] Li M., Liu G. H. & Izpisua Belmonte J. C. Navigating the epigenetic landscape of pluripotent stem cells. Nat Rev Mol Cell Biol 13, 524–535, doi: 10.1038/nrm3393 (2012). 22820889

[b2] Niwa H. How is pluripotency determined and maintained? Development 134, 635–646, doi: 10.1242/dev.02787 (2007). 17215298

[b3] van der Stoop P. *et al.* Ubiquitin E3 ligase Ring1b/Rnf2 of polycomb repressive complex 1 contributes to stable maintenance of mouse embryonic stem cells. PLoS One 3, e2235, doi: 10.1371/journal.pone.0002235 (2008). 18493325 PMC2375055

[b4] Endoh M. *et al.* Polycomb group proteins Ring1A/B are functionally linked to the core transcriptional regulatory circuitry to maintain ES cell identity. Development 135, 1513–1524, doi: 10.1242/dev.014340 (2008). 18339675

[b5] Ku M. *et al.* Genomewide analysis of PRC1 and PRC2 occupancy identifies two classes of bivalent domains. PLoS Genet 4, e1000242, doi: 10.1371/journal.pgen.1000242 (2008). 18974828 PMC2567431

[b6] Endoh M. *et al.* Histone H2A mono-ubiquitination is a crucial step to mediate PRC1-dependent repression of developmental genes to maintain ES cell identity. PLoS Genet 8, e1002774, doi: 10.1371/journal.pgen.1002774 (2012). 22844243 PMC3405999

[b7] Nassal* S. G. K. A. M. hRUL138, a novel human RNA-binding RING-H2ubiquitin-protein ligase. journal of cell science, doi: 10.1242/jcs (2003). 12538761

[b8] Zhou W. *et al.* Histone H2A monoubiquitination represses transcription by inhibiting RNA polymerase II transcriptional elongation. Mol Cell 29, 69–80, doi: 10.1016/j.molcel.2007.11.002 (2008). 18206970 PMC2327256

[b9] Nicassio F. *et al.* Human USP3 is a chromatin modifier required for S phase progression and genome stability. Curr Biol 17, 1972–1977, doi: 10.1016/j.cub.2007.10.034 (2007). 17980597

[b10] Joo H. Y. *et al.* Regulation of cell cycle progression and gene expression by H2A deubiquitination. Nature 449, 1068–1072, doi: 10.1038/nature06256 (2007). 17914355

[b11] Nakagawa T. *et al.* Deubiquitylation of histone H2A activates transcriptional initiation via trans-histone cross-talk with H3K4 di- and trimethylation. Genes Dev 22, 37–49, doi: 10.1101/gad.1609708 (2008). 18172164 PMC2151013

[b12] Zhang X. Y. *et al.* The putative cancer stem cell marker USP22 is a subunit of the human SAGA complex required for activated transcription and cell-cycle progression. Mol Cell 29, 102–111, doi: 10.1016/j.molcel.2007.12.015 (2008). 18206973 PMC2254522

[b13] Zhu P. *et al.* A histone H2A deubiquitinase complex coordinating histone acetylation and H1 dissociation in transcriptional regulation. Mol Cell 27, 609–621, doi: 10.1016/j.molcel.2007.07.024 (2007). 17707232 PMC2709280

[b14] Yang W. *et al.* The histone H2A deubiquitinase Usp16 regulates embryonic stem cell gene expression and lineage commitment. Nature communications 5, 3818, doi: 10.1038/ncomms4818 (2014). PMC4060806 24784029

[b15] Sussman R. T. *et al.* The epigenetic modifier ubiquitin-specific protease 22 (USP22) regulates embryonic stem cell differentiation via transcriptional repression of sex-determining region Y-box 2 (SOX2). J Biol Chem 288, 24234–24246, doi: 10.1074/jbc.M113.469783 (2013). 23760504 PMC3745368

[b16] Clague M. J., Coulson J. M. & Urbe S. Deciphering histone 2A deubiquitination. Genome Biol 9, 202, doi: 10.1186/gb-2008-9-1-202 (2008). 18226187 PMC2395231

[b17] Hammond-Martel I., Yu H. & Affar el B. Roles of ubiquitin signaling in transcription regulation. Cellular signalling 24, 410–421, doi: 10.1016/j.cellsig.2011.10.009 (2012). 22033037

[b18] Weake V. M. & Workman J. L. Histone ubiquitination: triggering gene activity. MolCell 29, 653–663, doi: 10.1016/j.molcel.2008.02.014 (2008). 18374642

[b19] Chen S., Choo A., Wang N. D., Too H. P. & Oh S. K. Establishing efficient siRNA knockdown in mouse embryonic stem cells. Biotechnology letters 29, 261–265, doi: 10.1007/s10529-006-9223-3 (2007). 17091374

[b20] Marks H. *et al.* The transcriptional and epigenomic foundations of ground state pluripotency. Cell 149, 590–604, doi: 10.1016/j.cell.2012.03.026 (2012). 22541430 PMC3398752

[b21] Ying Q. L. *et al.* The ground state of embryonic stem cell self-renewal. Nature 453, 519–523, doi: 10.1038/nature06968 (2008). 18497825 PMC5328678

[b22] Kondo T. *et al.* Polycomb potentiates meis2 activation in midbrain by mediating interaction of the promoter with a tissue-specific enhancer. Dev Cell 28, 94–101, doi: 10.1016/j.devcel.2013.11.021 (2014). 24374176

[b23] Schwartz Y. B. & Pirrotta V. Ruled by ubiquitylation: a new order for polycomb recruitment. Cell Rep 8, 321–325, doi: 10.1016/j.celrep.2014.07.001 (2014). 25061856

[b24] Tanay A., O'Donnell A. H., Damelin M. & Bestor T. H. Hyperconserved CpG domains underlie Polycomb-binding sites. Proc Natl Acad Sci USA 104, 5521–5526, doi: 10.1073/pnas.0609746104 (2007). 17376869 PMC1838490

[b25] Niederreither K. & Dolle P. Retinoic acid in development: towards an integrated view. Nat Rev Genet 9, 541–553, doi: 10.1038/nrg2340 (2008). 18542081

[b26] Hagege H. *et al.* Quantitative analysis of chromosome conformation capture assays (3C-qPCR). Nat Protoc 2, 1722–1733, doi: 10.1038/nprot.2007.243 (2007). 17641637

