# Supplementary material for: Dzip3 regulates developmental genes in mouse embryonic stem cells by reorganizing 3D chromatin conformation
Source: Sci Rep. 2015 Nov 16;5:16567. doi: 10.1038/srep16567 (PMC4645096; doi:10.1038/srep16567)
Supplement: Supplementary Information [file srep16567-s1.pdf]

Dzip3 regulates developmental genes in mouse embryonic stem cells by reorganizing 3D chromatin conformation

Daishi Inoue[1,2], Hitoshi Aihara[1], Tatsuharu Sato[3], Hirofumi Mizusaki[1], Masamichi Doiguchi[1], Miki Higashi[1], Yuko Imamura[1], Mitsuhiro Yoneda[1], Takayuki Miyanishi[4], Satoshi Fujii[5], Akihiko Okuda[6], Takeya Nakagawa[1*], Takashi Ito[1, 2*]

[1]Department of Biochemistry, Nagasaki University School of Medicine

[2]Nagasaki University Graduate School of Biomedical Sciences

[3]Department of Pediatrics, Nagasaki University Hospital

[4]Department of Environmental Science, Nagasaki University

[5] Kyushu Institute of Technology, Fukuoka820-8502, Japan

[6]Division of Developmental Biology, Saitama Medical School Research Center for Genomic Medicine

[*]Equal corresponding author

E-mail:tnakagawa@nagasaki-u.ac.jp, tito@nagasaki-u.ac.jp

Inoue et al. Supplementary Figure 1

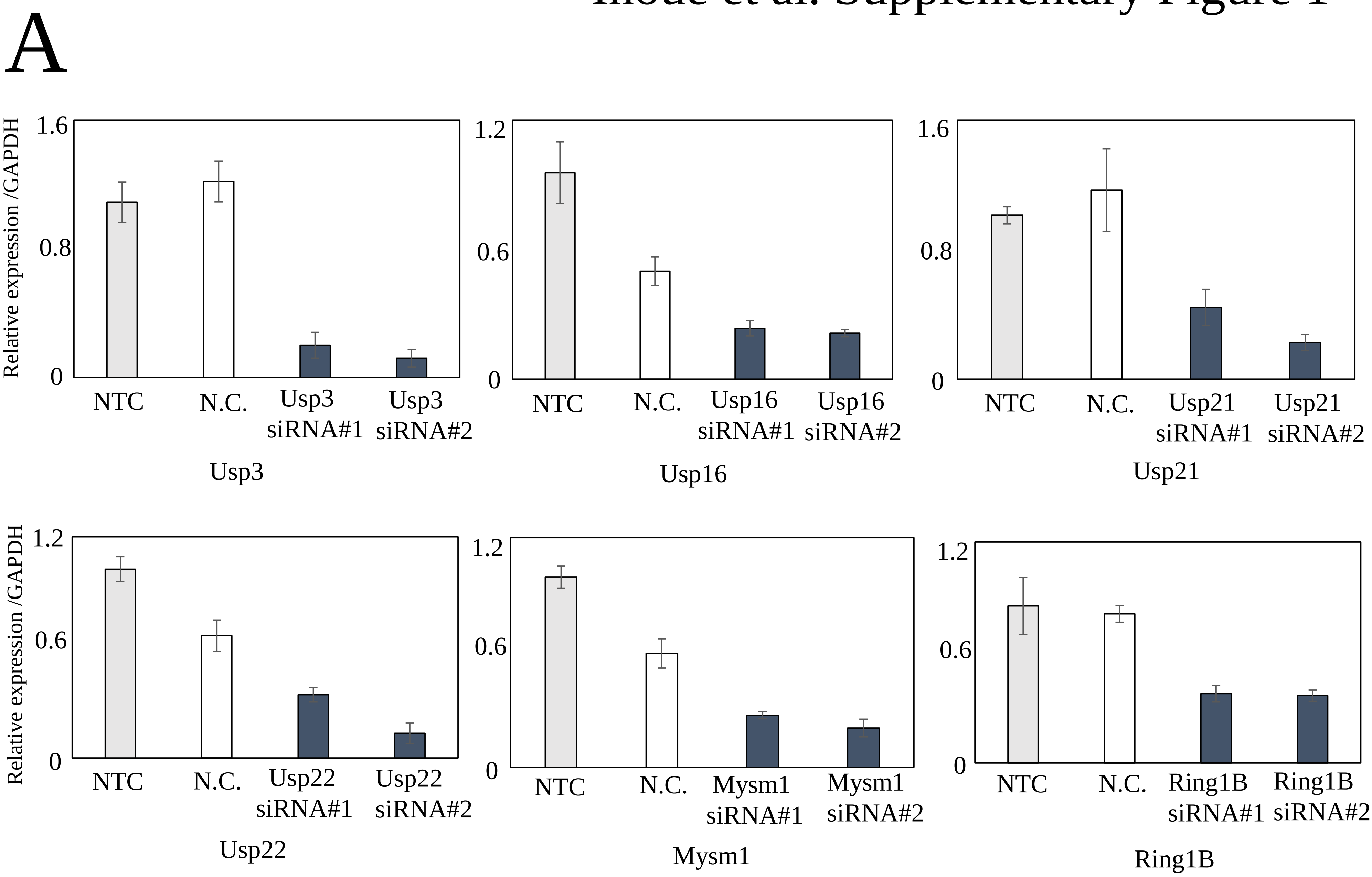

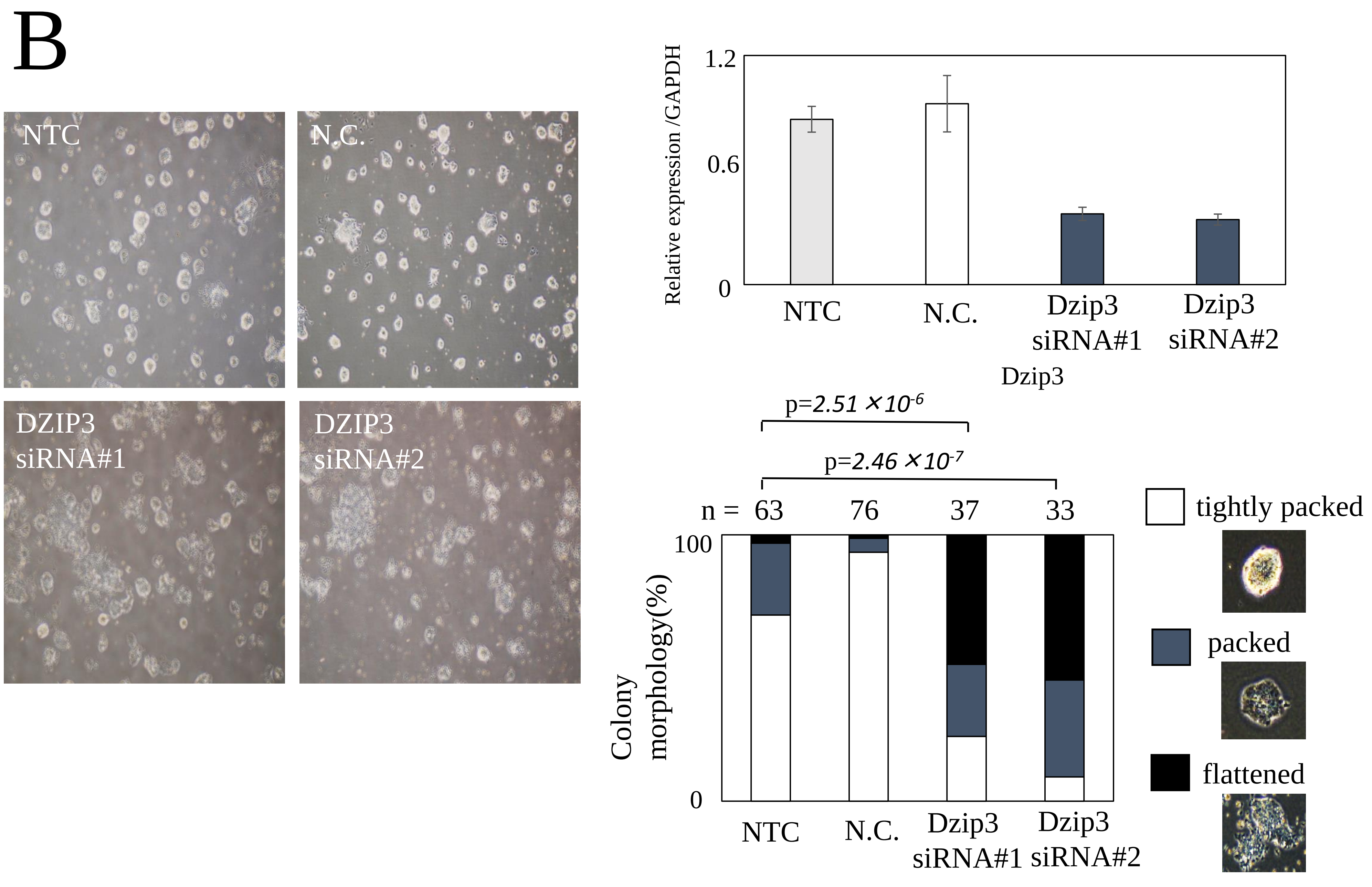

Inoue et al. Supplementary Figure 2

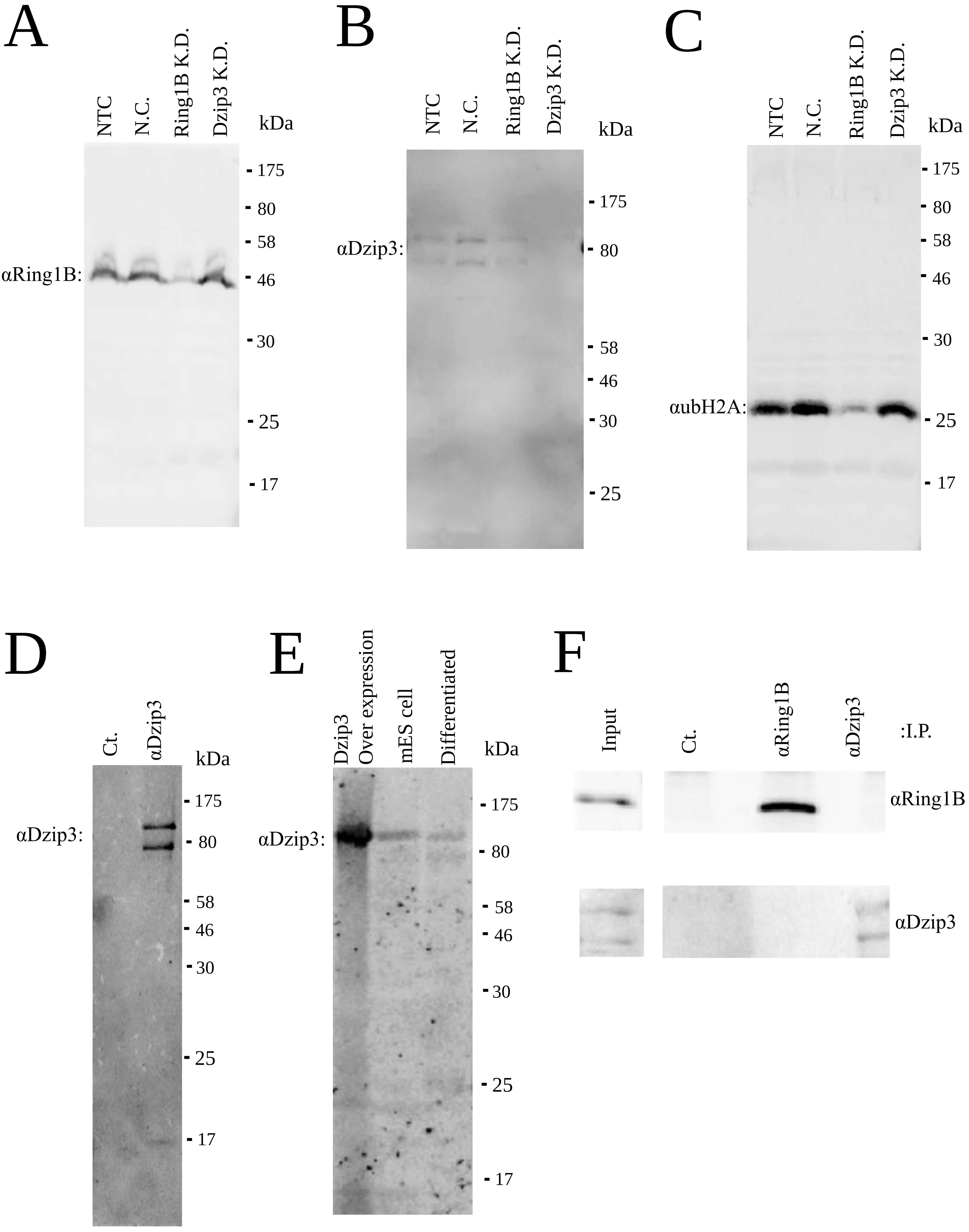

A

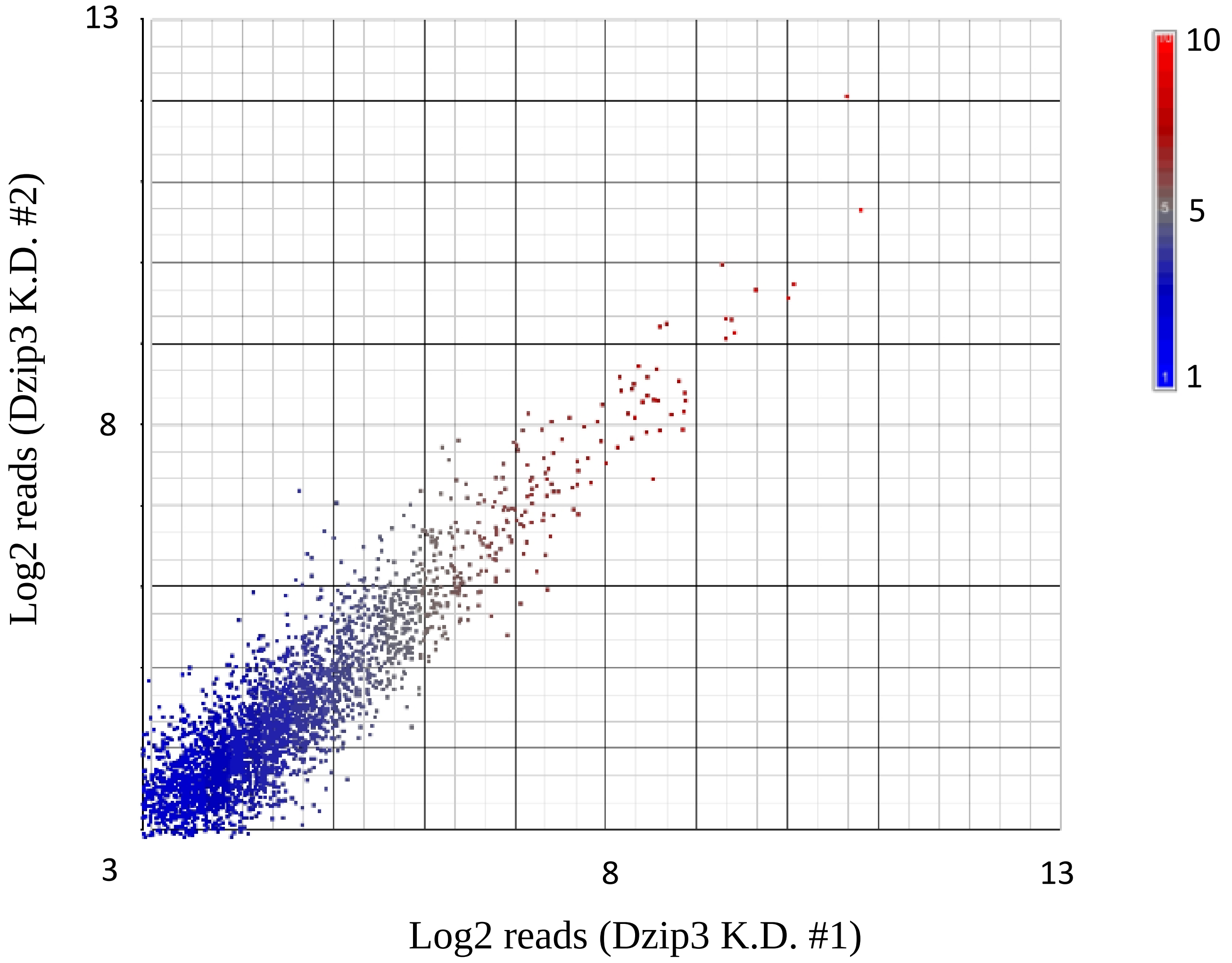
13
8
3
8
13
Log2 reads (Dzip3 K.D. #2)
Log2 reads (Dzip3 K.D. #1)
10
5
1

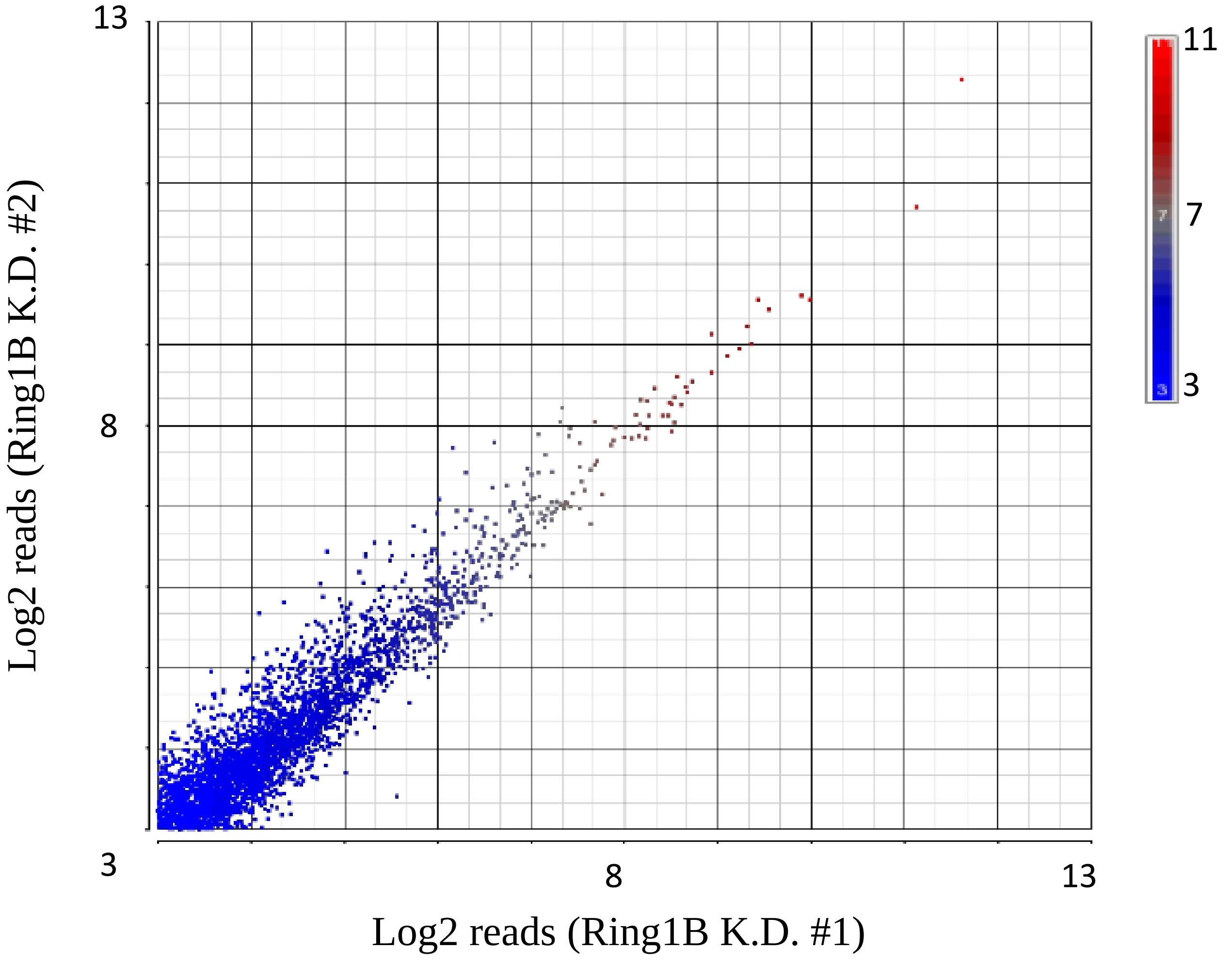
13
8
3
8
13
Log2 reads (Ring1B K.D. #2)
Log2 reads (Ring1B K.D. #1)
11
7
3

A

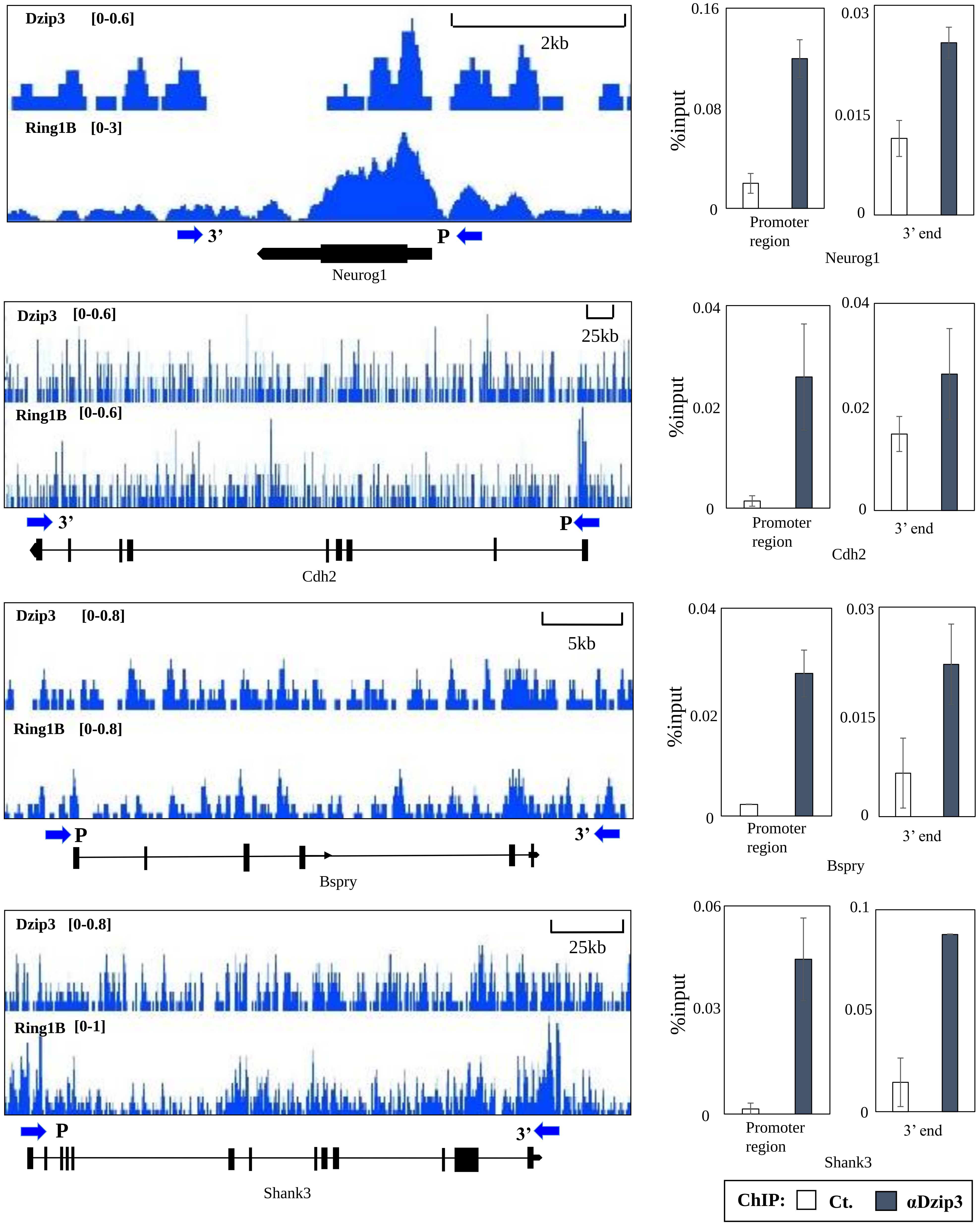
Dzip3 [0-0.6]
2kb
Ring1B [0-3]
3'
P
Neurog1
0.16
0.08
0
%input
Promoter region
0.03
0.015
0
3' end
Neurog1
Dzip3 [0-0.6]
25kb
Ring1B [0-0.6]
3'
P
Cdh2
0.04
0.02
0
%input
Promoter region
0.04
0.02
0
3' end
Cdh2
Dzip3 [0-0.8]
5kb
Ring1B [0-0.8]
P
3'
Bspry
0.04
0.02
0
%input
Promoter region
0.03
0.015
0
3' end
Bspry
Dzip3 [0-0.8]
25kb
Ring1B [0-1]
P
3'
Shank3
0.06
0.03
0
%input
Promoter region
0.1
0.05
0
3' end
Shank3
ChIP: Ct. αDzip3

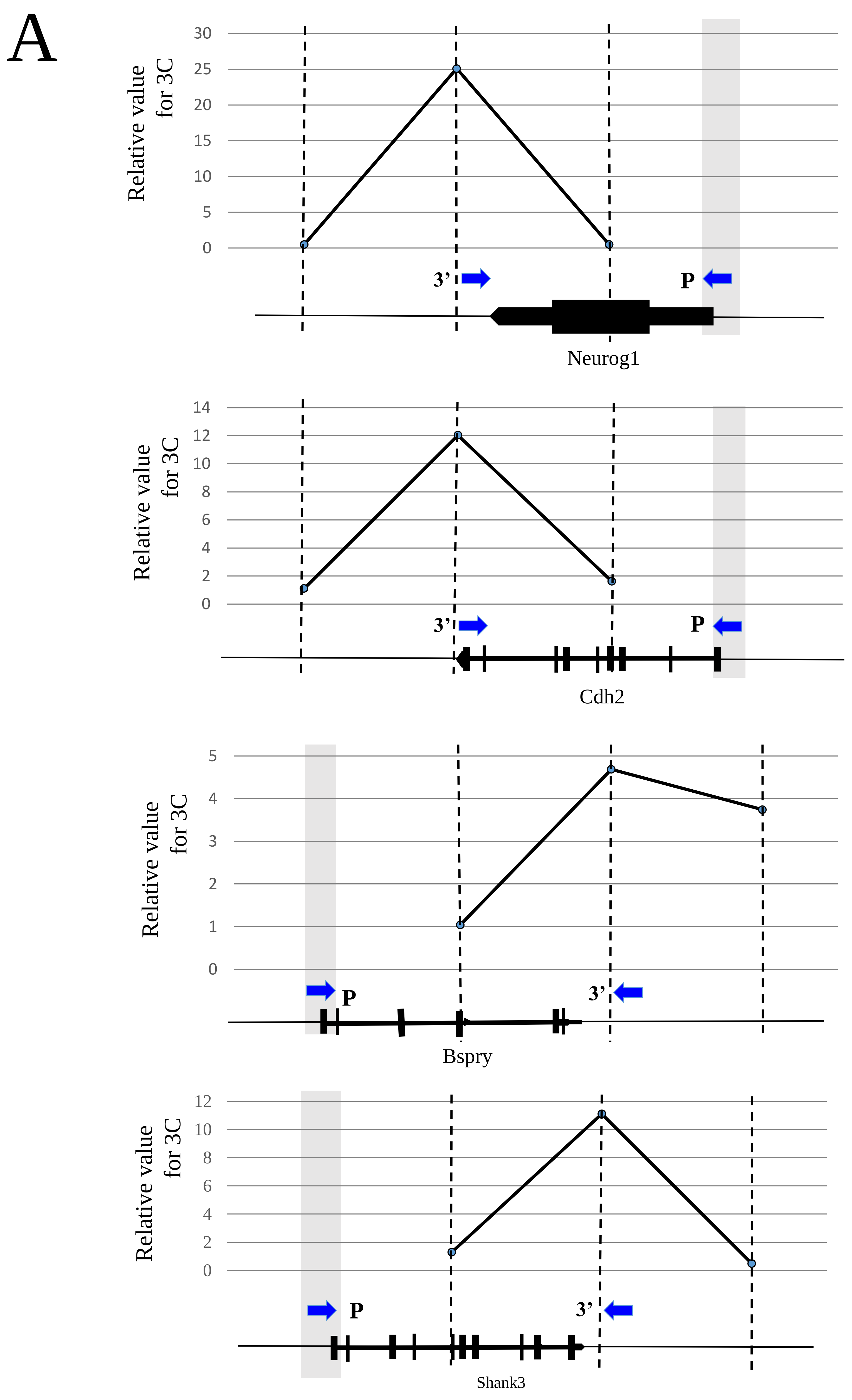
A
Relative value for 3C
30
25
20
15
10
5
0
3'
P
Neurog1
14
12
10
8
6
4
2
0
3'
P
Cdh2
5
4
3
2
1
0
P
3'
Bspry
12
10
8
6
4
2
0
P
3'
Shank3

Supplementary Table 1; siRNA sequence

USP3	MSS215066	AUACAACUCUGUCUCAUCAAGUUCC
	MSS215067	UAUCUAAGGAGAGAUCUAGGAAUGG
USP16	MSS232319	UAUCGCUGAUGUGAAACCACUGCCC
	MSS232320	UUCUGUUAGUGCCAGAUCAGGUGGC
USP21	MSS219991	AAGCAAUCCCGCAAAGACACUUUGC
	MSS282610	AGUACUACGUCUCAAAGUGGGUGGC
USP22	MSS211239	AUUGUCAUCACCUUUGCAGUGCCGG
	MSS211240	AAAUCGUUUGAGAUGGAAACAGGCC
Mysm1	MSS220542	UUCAUCGUCAGAUAACUUCUCAAUC
	MSS220543	AUUACAAGGUAUCAGUUGGAAAGGA
Ring1B	MSS208662	UUAAUUCACUGUGUAGACUUUCUAGG
	MSS276865	AACUCAUACAAACUGAGUUCCCAUG
Rnf8	MSS286125	AACCUUUGAUAUCAGCUGGUAUGUG
	MSS286127	AUGACUUCGUAUUCAUACUCCGCCG
Dzip3	MSS212594	AUUAUAUUCCAUCACAGGUGGACUC
	MSS212595	UUUCAAGGAAAGUUGGUCUUUAAGG

Supplementary Table 2; primer list

RT-qPCR		PCR product (bp)
GAPDH forward	AGCCTCGTCCCGTAGACAA	127
GAPDH reverse	ATGAAGGGGTCGTTGATGGC	
Dzip3 forward	CCCCAAGTCAGCACAAGGTT	187
Dzip3 reverse	GCATGTGCCCTGTTGCATCA	
Ring1B forward	AGGAAAGGGTCTTAGCAAGG	223
Ring1B reverse	TTGGTCCGTTTGTTACTCGG	
Oct 3/4 forward	CCTTGCAGCTCAGCCTTAAG	185
Oct 3/4 reverse	CAGAAACATGGTCTCCAGAC	
Sox2 forward	CAGCTACGCGCACATGAACG	212
Sox2 reverse	GCGAGTAGGACATGCTGTAG	
Nanog forward	TCGAATTCTGGGAACGCCTC	199
Nanog reverse	CCTTGTCAGCCTCAGGACTT	
Klf4 forward	ACTTGTGACTATGCAGGCTGT	106
Klf4 reverse	CGTCCCAGTCACAGTGGTAAG	
lefty1 forward	GGACAAGGCTGATGTGGAAGG	177
lefty1 reverse	CCGAACACTAGCAGGTGAGT	
T forward	CCAGCTCTAAGGAACCACCG	137
T reverse	ACTCCGAGGCTAGACCAGTT	
Stra8 forward	AGCATCCTTCAACCTGCAAGA	149
Stra8 reverse	CAACAGCCTCAGAGGGGAAC	
Eomes forward	CACAGTTCATCGCTGTGACG	178
Eomes reverse	CCAGGGACAATCTGATGGGA	
Acta1 forward	TACCACCGGCATCGTGTTG	190
Acta1 reverse	GCGCACAATCTCACGTTCAG	
Rhox6 forward	CTGGCTCAACTGCGGTACAG	154
Rhox6 reverse	ACCAATTCTGCACATCACATTCA	
Neurod1 forward	ACAGACGCTCTGCAAAGGTTT	163
Neurod1 reverse	GCACTGGTAGGAGTAGGGATG	
Neurog1 forward	GCGCTTCGCCTACAACTACAT	226
Neurog1 reverse	CGAGGGACTACTGGGGTCAG	
Cdh2 forward	AGGATGTGCACGAAGGACAG	101
Cdh2 reverse	CTTGAAATCTGCTGGCTCGC	
ChIP-qPCR (promoter region)		
Cdh2 forward	TCGCGGTGCGCGCG	176

Cdh2 reverse	TCCTGGCCACTTGGAGACTG	
Neurog1 forward	CGCGAGCATAAATTATGCAAATAGCAG	169
Neurog1 reverse	TGAGCACCAAGTGTGGCACA	
Neurod1 forward	ACAGGTCCGCGGAGTCTCTA	109
Neurod1 reverse	GAACCACGTGACCTGCCTAT	
Shank3 forward	GTGCAGTGGAAGGCAGTAGACA	169
Shank3 reverse	GCTGCGAGTGAACCAACTCAG	
Bspry forward	CAAGTATGAGAGTGAGATCGCCTCA	121
Bspry reverse	TGCTAAATGCATTAACAGCGTGTTTG	
ChIP-qPCR (3' end)		
Cdh2 forward	TGGACATCGCTGAAGACGCA	125
Cdh2 reverse	GCAGTGCTACCACCAAGCTACA	
Neurog1 forward	CTGCGCCACTGTGGCATCA	200
Neurog1 reverse	GCTCTTAGACTGGGGGAGGAAG	
Shank3 forward	CTGAGGTCATGTCCTACTGACTTGC	168
Shank3 reverse	TACCAGGAACTCATAAGTACCACCA	
Bspry forward	TGGACCAGTCTGATTGCAGGA	173
Bspry reverse	CATCTGACATGACACCCGTACTG	
3C-qPCR		
GAPDH (internal region) forwarc	CAATGTGTCCGTCGTGGATCTGA	
GAPDH (internal region) reverse	GCCTGCTTCACCACCTTCTTG	
Cdh2 (promoter region) forward	TGAGACCCACTCAGCTCCTGA	
Cdh2 (3' end) reverse	GGCGGGCAGTTTATTTGCAGC	
Cdh2 (3' end proximal region) re	GAAGCAGATCCTGTCAAGTGGAAAC	
Cdh2 (3' end distal region) rever	GTGCCCTGCTCACTGCCT	
Bspry (promoter region) forward	CAGCTACTGGCTCTTGAGTGCTTG	
Bspry (3' end) reverse	GTGGCTACCTTCCAGAAGGCT	
Bspry (3' end proximal region) re	ATCGACATCTTTGACACCAACCAT	
Bspry (3' end distal region) reve	CACTTGAGGCTGACAGGTGATGT	
Neurog1 (promoter region) forwa	AGTCAGTGTTCAGTTTGACGGAATG	
Neurog1 (3' end) reverse	GTGCAGCAACCTAACAAGTGGTC	
Neurog1 (3' end proximal region)	CTCCAGTCCAGTGCCTGAATAGC	
Neurog1 (3' end distal region) re	ATGTGAGGCTCTGCTGTAGCAAT	
Shank3 (promoter region) forwar	CTGTGTGGTAAGTGGTGTGTTGC	
Shank3 (3' end) reverse	CATCCAGGTCAGATGTCCTGGG	
Shank3 (3' end proximal region)	CTGGAGCAGGCAGGAACTGTG	
Shank3 (3' end distal region) rev	CGGCACCACTTGTTGCGT	

Supplementary Table 3; Miseq read number

		total read (reads passing filter)	mapped read (1st)	mapped read (2nd)
RNA-seq	mES cell Control #1	3295525	2864178	2849526
	mES cell Ring1B K.D. #1	3757563	3266171	3251234
	mES cell Dzip3 K.D. #1	3816009	3206851	3198805
	mES cell Control #2	3570604	3156740	3142344
	mES cell Ring1B K.D. #2	3549128	3122128	3108537
	mES cell Dzip3 K.D. #2	3900100	3431529	3425719
ChIP-seq	mES cell input	25047339	24167813	24132083
	mES cell Ring1B	26601407	25925601	25840353
	mES cell Dzip3	27439152	26675271	26598187
	mES cell ubH2A	34550852	33577225	33459604

Supporting Information

Supplementary Figure 1. Dzip3 knockdown with two independent siRNAs results in a decrease in the percentage of tightly packed cell colonies.

(a) The efficiency of knockdown was evaluated in mES cells (serum+LIF) transfected with the indicated specific and control siRNAs through RT-qPCR analysis. Values (normalized to the corresponding values of the internal control gene GAPDH) are the mean±SEM of three independent experiments. (b) Two independent Dzip3 siRNAs were used for transfection to exclude the possibility of off-target effects. Statistical significance was assessed by employing the HYPGEOMDIST function ($P=2.51\times10^{-6}$, negative control [NC] versus Dzip3 siRNA #1; $P=2.46\times10^{-7}$, NC versus Dzip3 siRNA #2). NTC, no-treatment control. Error bars, standard deviation.

Supplementary Figure 2. Full-length images of immunoblots and immunoprecipitation analysis.

(a)(b) Full-length images of blots corresponding to Figure 1B. (c) Full-length images of blots corresponding to Figure 1C. (d) Nuclear extracts were subjected to immunoprecipitation to confirm specificity of the Dzip3 antibody. (e) The specificity of the Dzip3 antibody was addressed by performing a western blot. (f) Immunoprecipitation was performed to exclude potential cross-reactivity of Dzip3 antibody with Ring1B antibody, as these proteins may serve unique or redundant roles in transcriptional regulation.

Supplementary Figure 3. The correlation between duplicate RNA-seq samples.

(a)Scatterplot of duplicate RNA-seq samples.

Supplementary Figure 4. ChIP-seq signal profile of target genes on a whole-gene scale and two-step cross-linking and ChIP-qPCR analysis

(a)Left panel: ChIP-seq signal profile of target genes on a whole-gene scale. Right panel: Two-step cross-inking and ChIP-qPCR analysis of the promoter region and the 3'end of the gene in control mES cells.

Supplementary Figure 5. 3C-qPCR analysis of long-distance interactions at the target gene locus.

(a)The relative level of each ligation product plotted according to its distance from the promoter P (see map below graphs). The data are normalized to a GAPDH loading control.

Supplementary Table 1. siRNA used in this study.

Supplementary Table 2. Primers used in this study.

Supplementary Table 3. The number of total and mapped reads in RNA-seq and ChIP-seq experiments.
